# Gaining new insights into nanoporous gold by mining and analysis of published images

**DOI:** 10.1038/s41598-018-25122-3

**Published:** 2018-04-30

**Authors:** Ian McCue, Joshua Stuckner, Mitsu Murayama, Michael J. Demkowicz

**Affiliations:** 1 Department of Materials Science and Engineering, Texas A&M University, College Station, TX 77843 USA; 2 Department of Materials Science and Engineering, Virginia Polytechnic Institute and State University, Blacksburg, VA 24061 USA

## Abstract

One way of expediting materials development is to decrease the need for new experiments by making greater use of published literature. Here, we use data mining and automated image analysis to gather new insights on nanoporous gold (NPG) without conducting additional experiments or simulations. NPG is a three-dimensional porous network that has found applications in catalysis, sensing, and actuation. We assemble and analyze published images from among thousands of publications on NPG. These images allow us to infer a quantitative description of NPG coarsening as a function of time and temperature, including the coarsening exponent and activation energy. They also demonstrate that relative density and ligament size in NPG are not correlated, indicating that these microstructure features are independently tunable. Our investigation leads us to propose improved reporting guidelines that will enhance the utility of future publications in the field of dealloyed materials.

## Introduction

**Figure 1:**
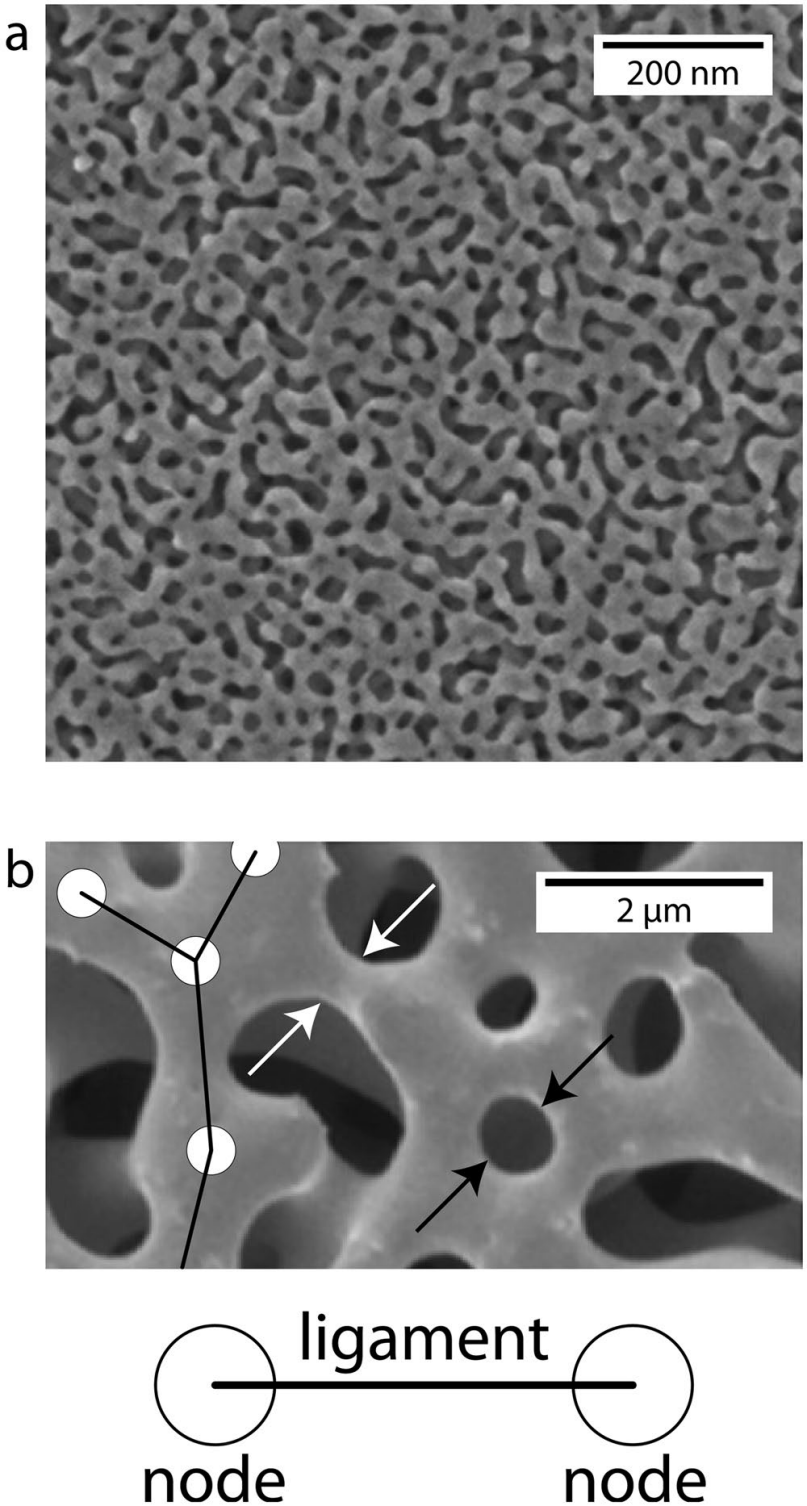
Nanoporous gold (NPG) and its physical characteristics. (**a**) Representative electron micrograph of NPG reprinted with permission from ref.^7^. (**b**) Higher magnification electron micrograph reprinted with permission from ref.^8^ illustrating some physical characteristics of NPG: nodes (shown as circles), ligaments (shown as lines), ligament diameter (white arrows), and pore diameter (black arrows).

Materials research and development is often frustratingly slow due to the time and resources needed to conduct new experiments^1^. However, it may be possible to accelerate materials development by systematically extracting new insights from already published literature using advanced image analysis tools. We apply this strategy to nanoporous gold (NPG): a material that has been studied extensively due its potential uses in catalysis^2,3^, sensing^4^, actuation^5^, and energy storage^6^. NPG consists of a network of interconnected nanometer-scale pores and ligaments, as shown in Fig. 1 ^7,8^. By mining and analyzing published, peer-reviewed, images such as those shown in Fig. 1, we obtain new insights into processing-structure-property relations in NPG without conducting any new experiments or simulations.

Our method relies on novel image-analysis software, developed and discussed in a previous publication^9^, to extract microstructure characteristics – such as NPG ligament and pore dimensions – in a consistent and reproducible manner. Combining this information with reported processing histories, we obtain a quantitative numerical description of NPG coarsening as a function of time and temperature. Our analysis confirms that coarsening in NPG is a thermally activated process, with an Arrhenius dependence on temperature and an activation energy consistent with surface self-diffusion of Au. However, our analysis finds a coarsening exponent that is lower than classical predictions developed for idealized systems, indicating a need for revised models to capture the coarsening behavior of dealloyed materials.

In addition, we find new insights concerning the relative density of NPG, approximated as the area fraction of the solid phase in published NPG images. Relative density is found not to correlate with other physical characteristics of NPG, such as ligament diameter, parent alloy composition, or processing conditions such as dealloying time or temperature. This finding suggests the existence of unreported, “hidden” processing parameters that may enable NPG relative density and ligament size to be independently tuned. Based on our investigation, we propose new publication guidelines that will facilitate the discovery of such unanticipated, hidden parameters from data mining studies on future publications in the field of dealloyed materials.

## Results

### Data Mining Approach and Analysis

NPG is a prototypical dealloyed material, formed by selectively dissolving Ag or Cu out of a parent Ag/Cu-Au alloy using an acid solvent^10^. It is an ideal target material for a data mining study such as ours because it has inspired a large volume of literature to analyze. Indeed, keyword searches on “dealloying” and “nanoporous” using the Web of Science database yield more than 1,500 and 22,000 hits, respectively. However, only a small fraction of these publications refers to work that uses de-alloying to synthesize NPG. An even smaller fraction contains the information needed for our study, such as high-quality images and a comprehensive description of material processing conditions. Since most widely used search engines do not have the capability to sort publications according to the type of data they contain, we confine our work to manuscripts citing the seminal paper on dealloyed NPG by Erlebacher *et al*.^11^. At the time of our search (03/01/2017), there were a total of 1293 citations for this paper listed on Web of Science. Out of these, only 145 contained sufficient information for our analysis. Of these 145 manuscripts, only 28 were focused on NPG^7,8,12–37^.

The automated image analysis software used in this study, AQUAMI^9^, applies advanced algorithms to extract accurate microstructural information even with significant additions of noise, blurring, and magnification errors (see Methods and Supplementary Fig. 1 for a schematic of the image analysis process). We used it to determine the mean ligament diameters (from a fitted Gaussian distribution), lengths, and solid phase area fractions from images published in the 28 manuscripts identified for further analysis for a total of 72 data points. The processing parameters of interest were: parent alloy composition; dealloying time and temperature; electrolyte solution and concentration; applied potential (if any); and coarsening time and temperature (if any). A compilation of the mined and analyzed data along with the corresponding references may be found in supplemental information. The following analysis includes mined data from both CuAu (9 data points) and AgAu (63 data points) alloys. We examined the mined data set with and without the CuAu samples, and found that the differences in our calculations were marginal and did not alter our conclusions. For completeness, both values are included in Supplemental Tables 1 and 2.

### Range of processing conditions and data quality

**Figure 2:**
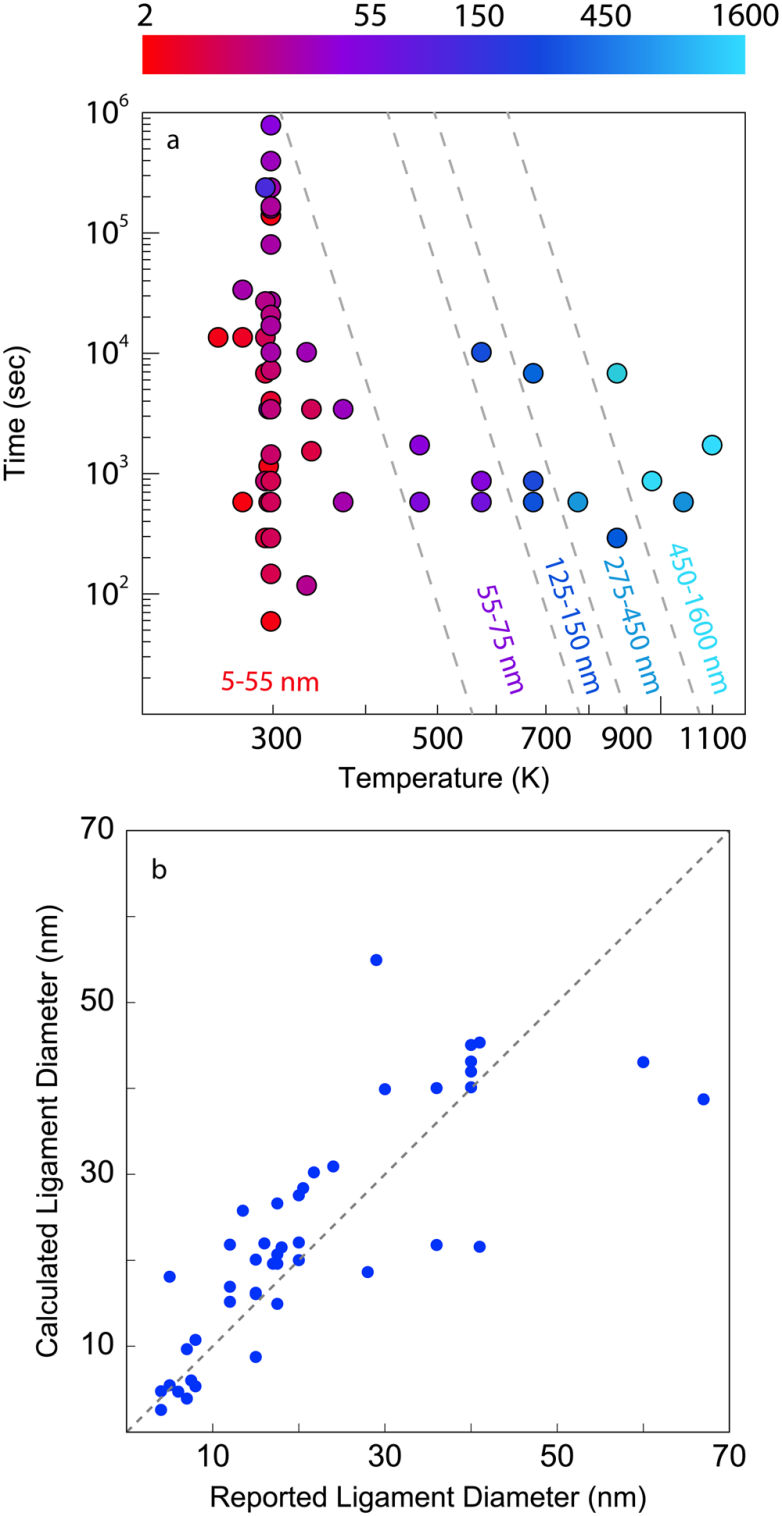
Assessment of data mined from the literature. (**a**) Map of processing conditions. Data points are color coded by the ligament diameter, ranging from red for 2.6 ± 1.3 nm to light blue for 1630 ± 770 nm. The dashed lines are approximate isocontours of the ligament diameter. (**b**) Calculated ligament diameters plotted against reported ligament diameters. The plot excludes six data points – with reported ligament diameters of 100, 140, 200, 480, 589, and 940 nm – to clearly compare data in the 2–70 nm range.

Figure 2a plots the range of coarsening times and temperatures reported in the publications mined in the present study. Each processing condition is represented as a single data point colored according to the calculated ligament diameter, *λ*, ranging from red at the smallest value (2.6 ± 1.3 nm) to light blue at the largest (1630 ± 770 nm), where ± is not an uncertainty but instead one standard deviation. Figure 2a shows that researchers have largely focused on room temperature coarsening across a wide range of times (60–864,000 sec) and short coarsening studies (durations less than 1200 sec) across a wide range of temperatures (253–1173 K). The dashed lines are approximate isocontours of *λ*, indicating that coarsening for long times at low temperatures may yield similar ligament diameters as short anneals at elevated temperatures. For example, room temperature coarsening for 864,000 seconds gives rise to a ligament diameter of 56 ± 20 nm, comparable to the ligament diameter produced by coarsening at 473 K for 600 sec, 63 ± 16 nm.

The literature used in our study reports ligament diameters for approximately 70% of the data points shown in Fig. 2a. We compare these published values with the ones we obtained using AQUAMI by plotting them against each other in Fig. 2b. If these ligament diameter pairs were equal, then all the data points plotted in Fig. 2b would lie on the diagonal dashed line shown in the figure. The high degree of scatter about this line indicates frequent discrepancies between the reported ligament diameters and ones determined by our image analysis.

We attribute these differences to approximations made in the publications mined for our study. For example, some authors measured only a handful of representative ligament diameters to estimate the ligament diameter of their samples^19,29,34,38^ and only three manuscripts employed computer-aided software to measure the ligament diameter^21,36,37^. Some authors reported using fast Fourier transform (FFT) image analysis to measure the ligament diameter under the assumption that ligament and pore diameters are equivalent^12,21^. However, this assumption does not accurately represent most NPG samples. Many manuscripts, moreover, failed to report the number of manual measurements used to determine the ligament diameter and often reported values without quantifying uncertainty (see Supplementary Materials)^7,13,14,16,18,23,25,28,30–32,35,37^. By contrast, AQUAMI analyzes all the ligaments in each image using automated image analysis to determine *λ* as well as its variance.

In addition to random scatter, some systematic discrepancies are also evident in Fig. 2b. Notably, more than 75% of the reported values are smaller than the ones obtained by image analysis. Indeed, one group of authors reported determining ligament diameters by measuring the thinner ligament regions mid-way between nodes, which would result in consistent underestimates of ligament diameter^38^. It is likely that similar choices were also made by other groups. AQUAMI avoids such approximations by determining ligament diameters over the entire ligament length, and allows us to examine the literature data in a consistent manner.

### Coarsening of NPG

NPG is known to undergo thermally-driven coarsening, manifested as a continuous increase in ligament diameter with time at rates that rise with temperature^39^. The data we mined for our study allow us to determine quantitative descriptions of this coarsening behavior. We expect that coarsening of NPG depends on one dominant mass transport mechanism, so— following Herring’s analysis^40^ – we fit a general power-law expression for the ligament diameter:

1 $$\lambda ={(kt{D}_{s})}^{n}.$$

Here, *t* is coarsening time excluding the time spent in dealloying (see data in supplemental materials), *D*_*s*_ is surface self-diffusivity, *n* is a coarsening exponent, and *k* is a proportionality constant. *D*_*s*_ has the usual Arrhenius form, ${D}_{s}={D}_{0}\exp [\,-\,{E}_{a}/{k}_{b}T]$, where *E*_*a*_ is the activation energy for rate-limiting process of coarsening. Thus, Eq. 1 may be rewritten as

2 $$\lambda =A{t}^{n}{e}^{-n{E}_{a}/{k}_{b}T},$$

where $A={(k{D}_{0})}^{n}$ collects all temperature- and time-independent proportionality constants.

**Figure 3:**
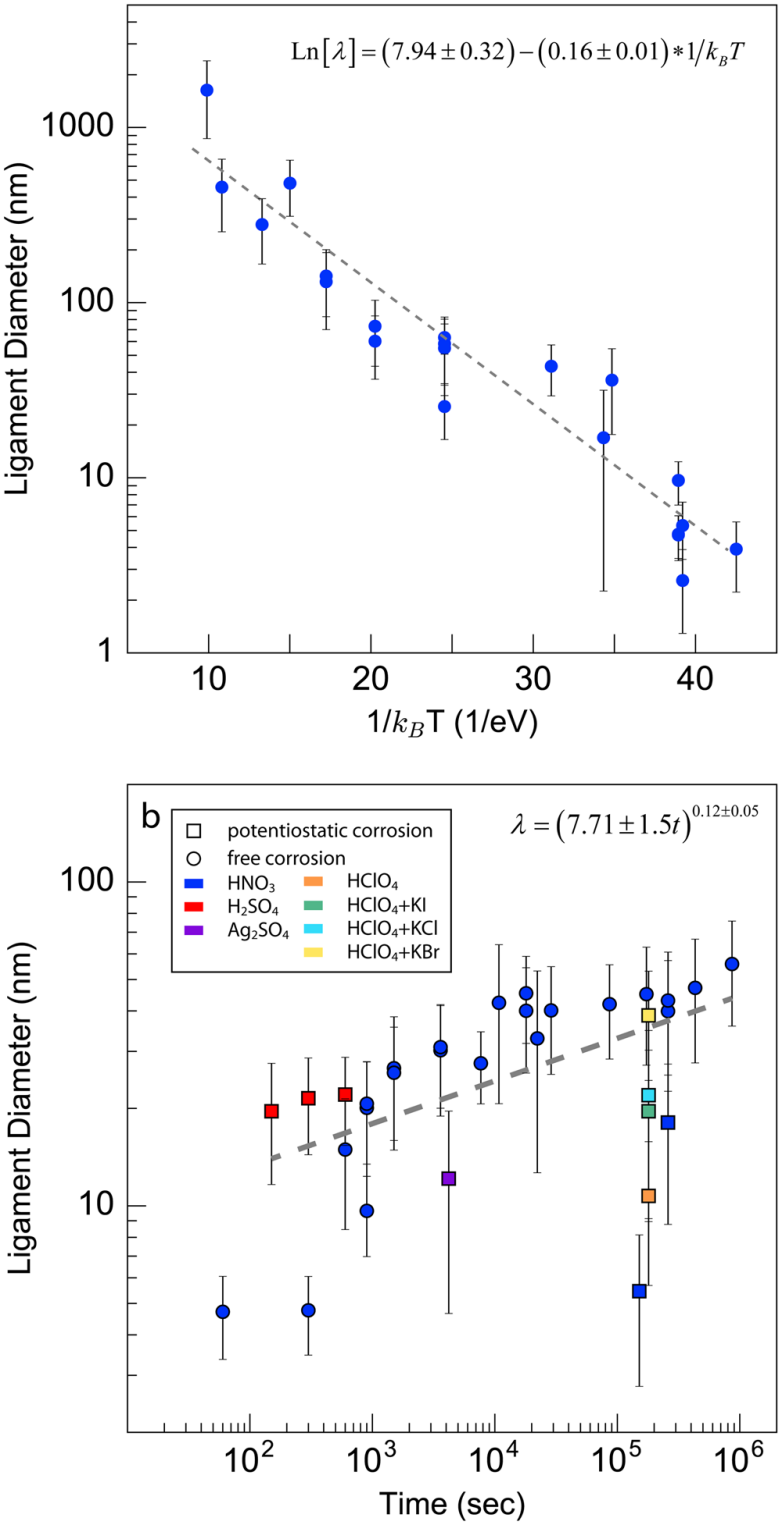
Coarsening of nanoporous gold. (**a**) Arrhenius plot of the ligament diameter after coarsening at elevated temperatures for short durations (600–1200 seconds). The dashed line is a linear least squares fit, whose slope equals −nE_a_ and whose intercept equals Ln[(*kD*_0_)]^*n*^. (**b**) Ligament diameter versus time for room temperature coarsening over a wide range of times. The dashed line is a linear least squares fit, whose slope equals n.

The data shown in Fig. 2a enables us to determine all of the parameters entering into Eq. 2, providing a full description of NPG coarsening. To this end, we break down the ligament diameter data into two groups. The first sweeps over a wide range of temperatures while retaining a fixed, narrow time window of 600–1200 sec. The Arrhenius plot of this data shown in Fig. 3a is consistent with thermally activated behavior^41,42^. Note that, according to Eq. 2, the slope of the best-fit line to the data in Fig. 3a—i.e., 0.16 ± 0.01 eV—corresponds to *nE*_*a*_ and not *E*_*a*_.

The second data group includes ligament diameters at a single temperature (room temperature) from a wide range of times. Plotting this data in Fig. 3b, we see a clear power-law dependence in time with best-fit exponent n = 0.12 ± 0.05 ≈ 1/8. Finally, using the values of n and *nE*_*a*_, we determine $A=3800\pm 750\,{\rm{cm}}\ast {\sec }^{-{\rm{n}}}$ for coarsening at room temperature in an electrolyte solution. The majority of the data in Fig. 3b originate from NPG synthesized *via* free corrosion (no applied potential) in nitric acid. However, some of the data are obtained from NPG fabricated using electrochemical corrosion (with an applied potential) or other electrolytes. Data from all processing conditions were included when fitting the exponent.

Combining the power-law exponent obtained from Fig. 3b with the slope of the Arrhenius plot in Fig. 3a, we calculate an activation energy of 1.33 ± 0.56 eV for the physical process governing NPG coarsening in air. This value falls approximately in the middle of the range of previously reported activation energies for surface self-diffusion of Au in air: 0.73 − 1.73 eV^41,42^. It should be noted that our activation energy calculation is not for the surface self-diffusion of Au in electrolytes, which is typically lower at ~0.6 eV^43^. Finally, using the average coarsening time of the samples in Fig. 3a, 660 seconds, Eq. 2 yields $A=1288\pm 500\,{\rm{cm}}\ast {\sec }^{-{\rm{n}}}$ for coarsening in air. This prefactor has approximately one-third the value for coarsening in any electrolyte concentration, indicating that A is sensitive to environmental conditions.

### NPG Relative Density

**Figure 4:**
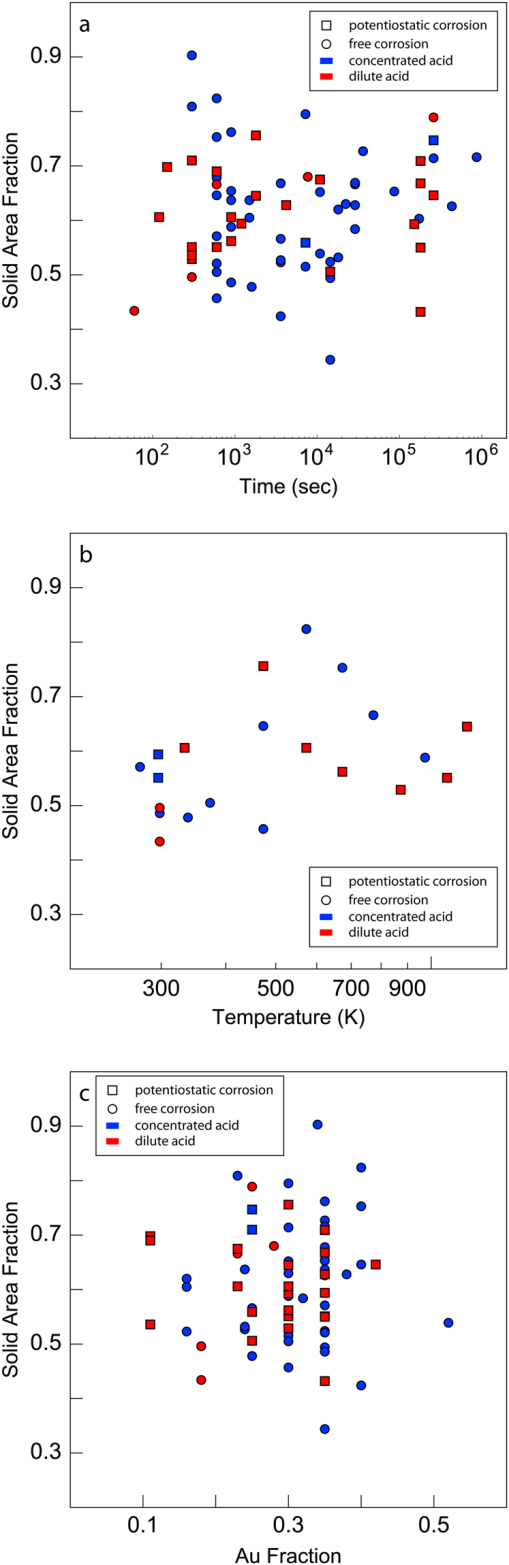
Lack of correlation between NPG area fraction and NPG processing parameters. (**a**) Coarsening duration at room temperature, (**b**) coarsening temperature for short durations (600–1200 seconds), and (**c**) gold fraction in the parent alloy.

Relative density is a key characteristic for predicting the properties of porous materials^44^. Although analysis of 2D images cannot give a direct measurement of relative density, we may nevertheless use the area fraction of the solid (gold) phase in NPG images as a convenient proxy for relative density. To determine the factors that control relative density, we sought to correlate solid phase area fractions for all the images mined in our study with NPG processing parameters, such as coarsening time and temperature, free vs. potentiostatic dealloying conditions, dilute (less than or equal to 0.1 M) vs. concentrated acid solvent (greater than 0.1 M), as well as the composition of the parent alloy. As shown in Fig. 4, however, the solid phase area fraction is not correlated to any of these parameters. Because the range of solid area fractions we found is very wide—spanning from 0.3 to 0.9—the lack of correlation cannot be due to inadequate sampling of the NPG relative density space. We therefore conclude that NPG relative density is controlled by a “hidden” processing parameter: one that is not sufficiently documented in the published literature to be uncovered *via* data mining.

**Figure 5:**
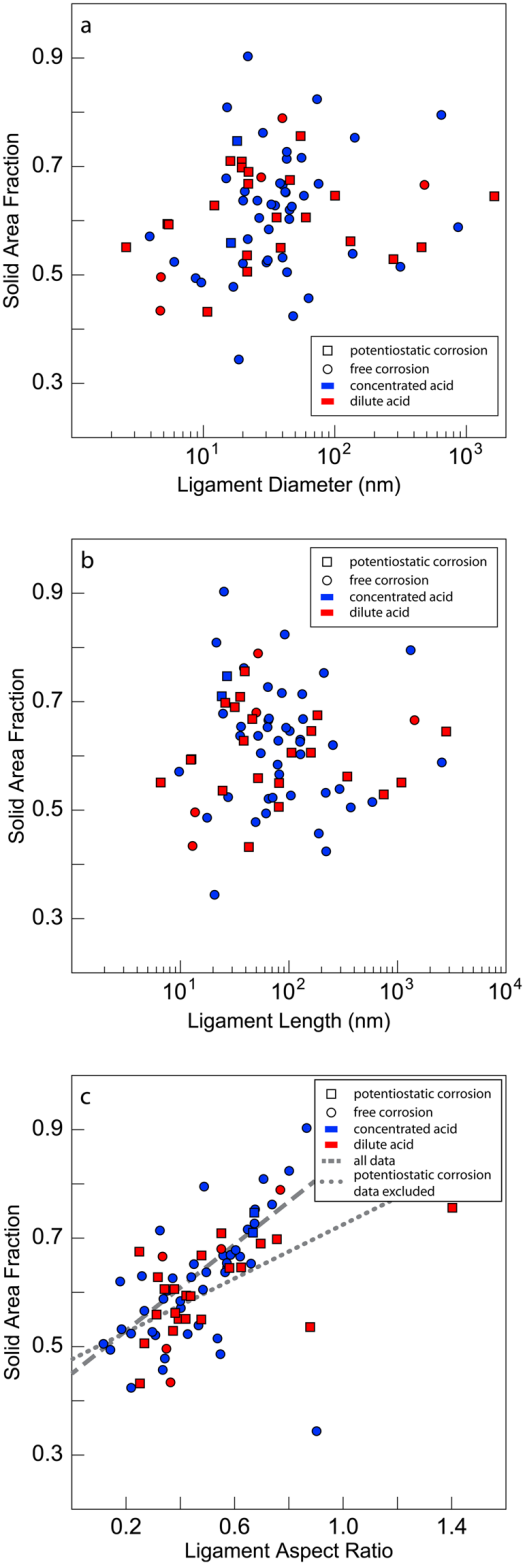
Area fraction versus morphological descriptors of NPG. (**a**) Ligament diameter, (**b**) ligament length, and (**c**) ligament aspect ratio (ligament diameter/ligament length). The lines in (**c**) are a linear least squares fits to the data. While there is no apparent correlation between the area fraction and either the ligament diameter or ligament length, there is a linear correlation between the solid area fraction and ligament aspect ratio.

To gain further insight into the factors controlling NPG relative density, we search for correlations between solid phase area fractions and other descriptors of NPG morphology, as shown in Fig. 5. We find no correlation with ligament diameter or ligament length. However, there is a clear proportionality between solid area fraction and ligament aspect ratio, defined as ligament diameter divided by ligament length. We interpret this outcome as evidence of “topological equivalence” among all the NPG images that we analyzed, i.e., that the interconnectivity of all ligaments is the same in all the NPG samples, regardless of processing method, degree of coarsening, or relative density. Under this assumption, any increase in relative density of an NPG sample must be achieved through the thickening of its ligaments, relative to ligament lengths. This interpretation is consistent with the observed correlation of relative density and ligament aspect ratio. It also supports the hypothesis that NPG coarsens and densifies in a topologically self-similar manner^39,45–47^.

## Discussion

In this study, we demonstrated that mining and analysis of published images is an effective way to gain new insight into processing-structure-property relations in materials. Applying this approach to NPG, we confirmed that coarsening is thermally activated in this material and calculated activation energies consistent with surface self-diffusion of Au being the rate-limiting process for coarsening. We also determined the coarsening exponent to be ~1/8. The strong correlation of ligament diameter with time and temperature demonstrates that they are the primary factors influencing coarsening, but our analysis may become more precise if we are able to include secondary and tertiary factors such as average grain size and defect densities. In addition, we find that NPG relative density, represented by solid phase area fraction, is not correlated with any of the processing conditions reported in the literature mined for our study. Furthermore, while solid area fraction is not correlated to ligament length or diameter, it shows a distinct correlation with ligament aspect ratio, supporting the notion that all the NPG images we investigated are topologically equivalent.

Our findings have important consequences for future investigations of NPG. First, Fig. 2 exhibits regions of NPG processing space that have remained unexplored, highlighting opportunities for future studies. In particular, there are no reported investigations for short coarsening times (less than 300 sec) at elevated temperatures (400–1300 K) and long coarsening times (greater than 900 sec) at intermediate temperatures (400–600 K). Additionally, Fig. 3 shows that during dealloying under potentiostatic conditions, or in different anion solutions, ligament diameters exhibit deviations from the main trend obtained for free corrosion in nitric acid, indicating a need for systematic investigations of the effect of dealloying potential and solvent chemistry on NPG morphology. The effects of applied potential have been examined in ref.^48^ and there has been one in-depth study regarding the dealloying potential, volume shrinkage, and remaining Ag content^49^. Unfortunately, we were unable to extract data from ref.^48^ due to poor image contrast while ref.^49^ provided no images corresponding to the 36 reported processing conditions.

The coarsening exponent of n ≈ 1/8 obtained in our study stands in contrast with the classical surface diffusion exponent of 1/4, which was derived for the idealized case of a sinusoidal surface profile decaying by surface diffusion^50^. However, several key assumptions – particularly that surface diffusion is isotropic and the surface profile remains sinusoidal as it decays – are not expected to hold during coarsening in nanoporous gold. Departures from classical behavior have been observed in materials with finite terrace widths below the roughening transition temperature^51,52^. In addition, kinetic Monte Carlo coarsening studies of NPG by Erlebacher showed that a power-law exponent of *n* = 1/4 is only observed at long times, when the morphology approaches that of a sphere. Our findings indicate that the NPG samples investigated in the literature mined for our study are still far from this limiting condition. Had we used an exponent of *n* = 1/4 in our analysis, we would have obtained estimates of 0.64 ± 0.04 eV for the activation energy of Au surface self-diffusion, which is out of the range, 0.73–1.73 eV, of reported activation energies in the literature^41,42^.

There are a few studies on coarsening of NPG reported in the literature, but the results are inconclusive^12,53–56^. Reported coarsening power-law exponents have ranged from 0.13^54^ to 0.32^56^. One coarsening study^55^ did not directly report a coarsening relationship, but showed that their data was poorly captured by power-law exponents of 1/3 and 1/4. Only ref.^54^ measured ligament diameters directly from images, yielding a value close to that determined in our present study. The other manuscripts estimated the ligament diameter from scattering peaks corresponding to a characteristic length scale in the material under the assumption that ligament and pore diameters are identical. This assumption does not always hold, and there are appreciable differences in our calculated values and those reported in ref.^12^. Regarding the activation energy for coarsening, ref.^12^ reported a value of ~0.65 eV in an electrolyte, and is thus not comparable to our study, while ref.^53^ reported an unphysically low value of ~0.35 eV in air. Ref.^56^ did not directly report an activation energy, but showed that the data was better fit by a value of 0.64 eV than 2.2 eV. Of these three studies, we can only draw direct comparison with ref.^53^, but the value reported in that study corresponds to *nE*_*a*_ and not *E*_*a*_.

The prefactor *A* in the coarsening law for NPG (Eq. 2) appears to be highly sensitive to the coarsening conditions. For example, the value of *A* determined for coarsening in nitric acid is nearly a factor of three larger than for coarsening in air. *A* collects temperature- and time independent quantities that represent the morphology and topology of the coarsening NPG, the arrangement of Au surface lattice sites, as well as atomic jump distances and attempt frequencies during surface diffusion. As stated above, we expect that all the NPG images we analyzed are topologically equivalent. Thus, barring any major changes in surface structure, the fact that A has a higher value in an electrolyte than in atmosphere may be due to an elevated effective attempt frequency for surface diffusion, giving rise to increased *D*_0_. In the context of this interpretation, the difference in A between concentrated and dilute electrolytes is unexpected, since it suggests a marked sensitivity of attempt frequencies to the exact electrolyte composition.

Our study shows no correlation between NPG relative density and parent alloy composition. The formation of NPG during dealloying is normally presumed to involve near complete removal of Ag from the parent alloy^11^. The self-organization of the remaining Au into a morphology such as that shown in Fig. 1 is thought to occur through a surface diffusion process that conserves lattice sties^11^. If both these assumptions hold true, then a direct correlation between relative density and parent alloy composition is expected, contrary to the outcome of our analysis. Our finding therefore implies either that a significant portion of Ag in fact remains in solution upon dealloying or that the dealloying process does not conserve lattice sites.

Our findings also carry important implications for synthesis and processing of NPG. Ligament diameters are well-modeled by the analytical coarsening law stated in Eq. 2, indicating that little may be done to influence them beyond adjusting the dealloying time and temperature. However, NPG relative density shows no correlation with the ligament diameter, suggesting that these two features are in fact independent and may be adjusted separately. The ability to tune NPG relative density and ligament diameter independently of each other is of great interest for NPG development, as it widens the design space to optimize material properties such as strength, ductility, or toughness. Unfortunately, the information reported in the literature on NPG is inadequate to discover the processing parameters that govern NPG relative density.

One possible candidate for such a “hidden” parameter is the dissolution rate of Ag from the parent alloy. This parameter affects the two factors relevant to NPG relative density: a) the remaining Ag content in the parent alloy upon completion of dealloying and b) the extent of sample shrinkage (and consequently reduction in number of lattice sites) during dealloying. Unfortunately, neither Ag dissolution rate, nor final Ag content, nor sample shrinkage are consistently reported in the literature, even though individual studies show volume shrinkage may be as large as 30%, in some cases^57^.

The conclusions of the study presented here depend on the availability of data in the open literature, and our investigation reveals serious challenges in extracting this data. A surprisingly large number of the manuscripts we considered, ~89%, did not meet the minimum criteria to be used in our study due to poor image contrast, low image resolution, or lack of detailed processing history. Even if a manuscript met the minimum criteria, reported ligament diameters frequently had no corresponding images: a significant concern in light of the discrepancies between the reported and calculated ligament diameters shown in Fig. 2b. Increasing the number of high-quality images will lead to improved confidence in our analysis. However, it should not be overlooked that our automated software performed of order 1,000 measurements per image, significantly advancing the accuracy of feature sizes reported in the literature. In addition, our analyzed data (included as Supplementary Information) can be seen as a repository for our current understanding of NPG processing.

To enhance the utility of future publications, we propose that the following data be included in every publication on NPG (as well as other materials processed by dealloying): high quality images with minimum resolution of 300 DPI and at least 10 pixels per ligament diameter; representative cross-section images (to allow an assessment of the effect of free surfaces on NPG microstructure); dealloying and coarsening times; dealloying and coarsening temperatures; electrolyte solution and concentration; applied potential and current relative density; composition of the parent and final dealloyed material (in particular, the final Ag content of the material) and percent volume change of the sample (e.g., measured as change in film thickness upon dealloying, when the parent alloy comes in the form of Au-Ag leaf); and finally, include all images as supplemental material whenever possible. Meeting these criteria does not require significant additional effort, given access to standard materials research equipment, such as a scanning electron microscope with elemental analysis capabilities. Although we are unaware of published data in the field of nanoporous metals demonstrating significant inaccuracies in using 2D over 3D images to gather quantitative structural information, there is evidence that 2D measurements are accurate in comparison to 3D measurements in metallic foams^58^. It would be useful to quantify this relationship in nanoporous metals and other complex structures due to the popularity of 2D analysis in metallic foams and other cellular materials^59^.

The data mining approach used in this manuscript is not confined to NPG. As noted in the Data Mining section, 116 additional manuscripts contained sufficient information for image analysis, but were not used in our study because they focused on dealloyed materials other than Au, such as Cu or Pt. Our approach is directly applicable to those materials, given sufficient data. More broadly, data mining and image analysis may be applied to study numerous materials-related phenomena, such as solidification, precipitation, and grain growth. To accelerate investigations such as ours, it would be helpful to develop techniques for the automatic acquisition and screening of images and data from the published literature.

## Methods

### Image analysis

The NPG images used in this study were exported in TIFF format from manuscripts using Adobe Illustrator without any reduction in image resolution. The images were analyzed using a custom segmentation and measurement procedure implemented in the AQUAMI software^9^. The segmentation procedure consists of two steps: first, bilateral filtering to remove noise from the micrographs while preserving edges; second, Local Otsu’s Method to assign pixels to the solid or void phase, generating a binary image. The measurement procedure consists of three steps: first, a distance map is generated where pixels belonging to the solid phase are replaced with a value equal to the pixel’s Euclidean distance to the nearest pixel belonging to the void phase; next, a binary array is generated comprised of one pixel-thick lines along the center of the solid phase in the distance map; finally, a radius map is generated by element-wise multiplication of the distance map and binary array. This procedure is able to output the area fraction and full ligament diameter distribution. A similar approach was used to measure the ligament length, where nodes were removed from the binary array and a connected-components labelling algorithm was used to determine the number of pixels in each ligament. Image analysis details may be found in ref.^9^.

### Data availability

All data generated or analyzed during this study are included in Supplementary Information files.

## Electronic supplementary material

Supplementary Information

Supplementary Dataset 1
