# Supplementary material for: Gaining new insights into nanoporous gold by mining and analysis of published images
Source: Sci Rep. 2018 Apr 30;8:6761. doi: 10.1038/s41598-018-25122-3 (PMC5928227; doi:10.1038/s41598-018-25122-3)
Supplement: Supplementary file 1 — Supplementary Information [file 41598_2018_25122_MOESM1_ESM.doc]

**Supplemental Information for:**

**Gaining new insights into nanoporous gold by mining and analysis of published images**

Ian McCue^1*^, Joshua Stuckner^2^, Mitsu Murayama^2^, Michael J. Demkowicz^1^

^1^ Department of Materials Science and Engineering, Texas A&M University, College Station, TX 77840

^2^ Department of Materials Science and Engineering, Virginia Polytechnic Institute and State University, Blacksburg, VA 24061

Corresponding author: [imccue1@tamu.edu](mailto:imccue1@tamu.edu)

**1. Supplementary Info**


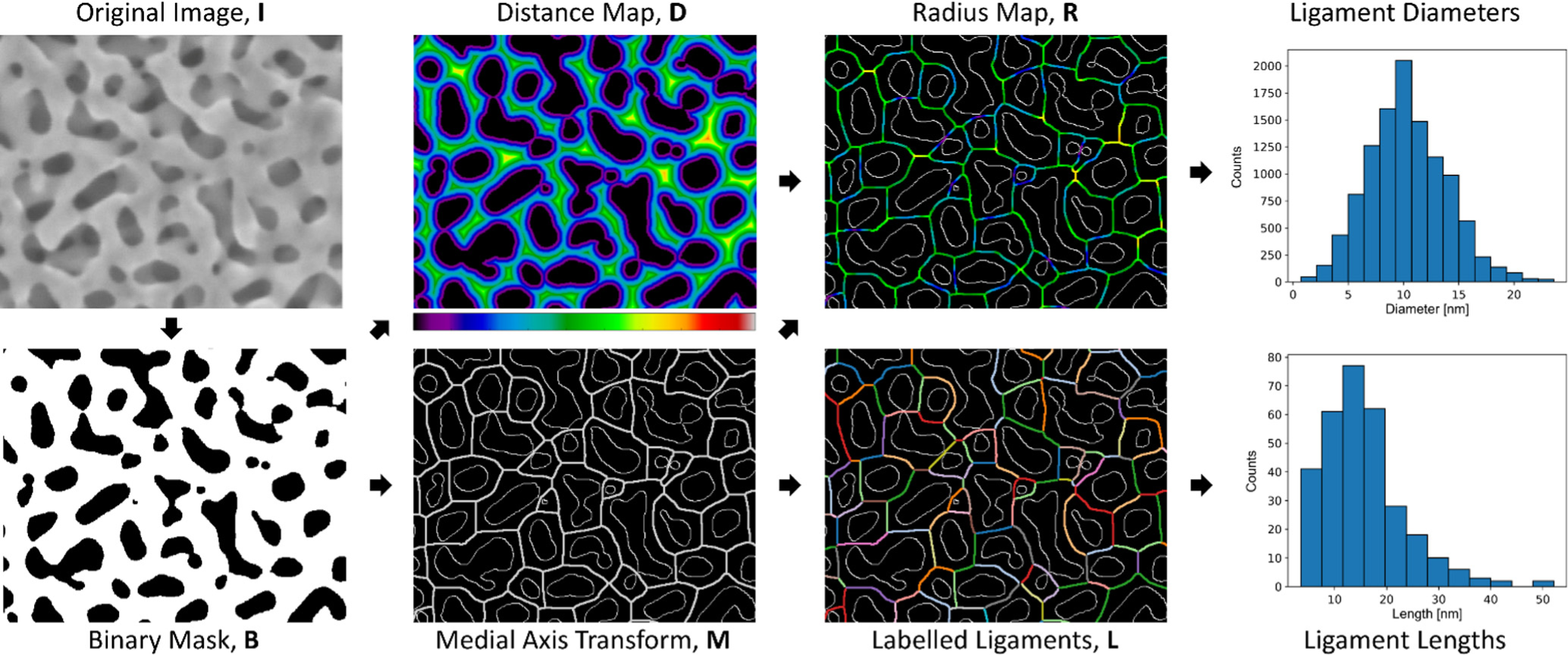


Supplementary Figure 1. Schematic of image analysis procedure used by AQUAMI software used in our study. First, a binary image is generated by noise removal *via* bilateral filtering, and assigned pixels to void (black) or solid (white) phases *via* Local Otsu’s Method. Second, a distance map is generated by measuring the pixel’s Euclidean distance to the nearest pixel belonging to the void phase. Third, a binary array is generated comprised of one pixel-thick lines along the center of the solid phase in the distance map. Finally, a radius map is generated by element-wise multiplication of the distance map and binary array. This procedure is able to output the area fraction and full ligament diameter distribution. A similar approach was used to measure the ligament length, where nodes were removed from the binary array and a connected-components labelling algorithm was used to determine the number of pixels in each ligament. Reprinted with permission from Ref. 9 of the manuscript.

**2. Supplementary Tables**

Supplementary Table 1. NPG Coarsening Parameters with Cu-Au alloy data.

| coarsening exponent | coarsening exponent in concentrated electrolytes | coarsening prefactor in concentrated and dilute electrolytes | coarsening prefactor in concentrated electrolytes | coarsening prefactor in air |
| --- | --- | --- | --- | --- |
| 0.12 ± 0.05 | 0.11 ± 0.05 | 3800 ± 750 $\text{cm}*\text{sec}^{\text{-n}}$ | 6100 ± 500$\text{cm}*\text{sec}^{\text{-n}}$ | 1288 ± 550$\text{cm}*\text{sec}^{\text{-n}}$ |

Supplementary Table 2. NPG Coarsening Parameters without Cu-Au alloy data.

| coarsening exponent | coarsening exponent in concentrated electrolytes | coarsening prefactor in concentrated and dilute electrolytes | coarsening prefactor in concentrated electrolytes | coarsening prefactor in air |
| --- | --- | --- | --- | --- |
| 0.13 ± 0.05 | 0.12 ± 0.05 | 3500 ± 690 $\text{cm}*\text{sec}^{\text{-n}}$ | 4800 ± 500$\text{cm}*\text{sec}^{\text{-n}}$ | 1380 ± 650$\text{cm}*\text{sec}^{\text{-n}}$ |
